# One-step approach for full-thickness skin defect reconstruction in rats using minced split-thickness skin grafts with Pelnac overlay

**DOI:** 10.1186/s41038-019-0157-0

**Published:** 2019-08-13

**Authors:** Tong Liu, Chao Qiu, Chi Ben, Haihang Li, Shihui Zhu

**Affiliations:** 1 Department of Burn Surgery, Institute of Burns, The First Affiliated Hospital, Naval Medical University, Shanghai, 200433 China; 2 Emergency Department, The First Affiliated Hospital, Naval Medical University, Shanghai, 200433 China

**Keywords:** Skin wound healing, Full-thickness skin defect, Minced skin graft, Pelnac, Split-thickness skin grafts, Reconstruction

## Abstract

**Background:**

Split-thickness skin grafting is the current gold standard for the treatment of traumatic skin loss. However, for patients with extensive burns, split-thickness skin grafting is limited by donor skin availability. Grafting split-thickness skin minced into micrografts increases the expansion ratio but may reduce wound repair quality. Dermal substitutes such as Pelnac can enhance the healing of full-thickness skin wounds, but their application currently requires two surgeries. The present study investigated whether it is possible to repair full-thickness skin defects and improve wound healing quality in a single surgery using Pelnac as an overlay of minced split-thickness skin grafts in a rat model.

**Methods:**

A full-thickness skin defect model was established using male Sprague-Dawley rats of 10 weeks old. The animals were randomly divided into control and experimental groups in which Vaseline gauze and Pelnac, respectively, were overlaid on minced split-thickness skin grafts to repair the defects. Wound healing rate and quality were compared between the two groups. For better illustration of the quality of wound healing, some results were compared with those obtained for normal skin of rats.

**Results:**

We found that using Pelnac as an overlay for minced split-thickness skin grafts accelerated wound closure and stimulated cell proliferation and tissue angiogenesis. In addition, this approach enhanced collagen synthesis and increased the formation of basement membrane and dermis as well as the expression of growth factors related to wound healing while reducing scar formation.

**Conclusions:**

Using minced split-thickness skin grafts overlaid with Pelnac enables the reconstruction of full-thickness skin defects in a single step and can increase the healing rate while improving the quality of wound healing.

## Background

As the first barrier against environmental insults, the skin has a vital physiological function. Large-area skin defects caused by burns and trauma can lead to pathogen invasion, dysregulation of body temperature, water loss from the body, and other serious consequences that endanger life [1–3]. It is therefore critical to repair skin defects quickly. Split-thickness skin grafting is the current gold standard treatment for significant traumatic skin loss, especially from burn injuries [4]. However, for burns spanning a large area, split-thickness skin grafting may be limited by the availability of donor skin. To overcome this problem, split-thickness skin grafts are typically minced into micrografts to increase the expansion ratio; however, this can compromise wound healing quality. In the case of dermal tissue defects, fibroblasts secrete immature extracellular matrix (ECM) into the wound area, leading to the formation of scars. A newly formed epidermis cannot establish close contact with dermal tissue, because a new basement membrane cannot be established; as a result of this unstable epidermal-dermal connection, the epidermis readily blisters and falls off, leading to chronic ulcers [5].

In the 1980s, the burn center at the Shanghai Ruijin Hospital carried out a clinical study of skin autografts combined with an allograft in a procedure known as intermingled transplantation. This technique involved wrapping a sheet allograft around the wound and punching holes about 1 cm apart, with 0.25-cm^2^ autograft pieces placed into the holes. A study of 12 patients who underwent this procedure documented the so-called sandwich phenomenon, whereby the skin autograft migrates between the dermis and epidermis of the allograft, causing it to degenerate as a result of host rejection while leaving the autograft intact. The allograft thus protects the autograft during the healing process. Since allogenic dermis is slowly degraded, it may serve as a scaffold to promote autologous dermal regeneration [6, 7].

The ideal particulate skin cover is allogenic skin, but it is difficult and costly to obtain. The next best option is fresh pigskin, but immune rejection and biosafety are major problems. Dermal substitutes such as Pelnac (Gunze Corp., Osaka, Japan) have therapeutic potential for the treatment of full-thickness skin wounds and enhance the quality of wound healing [8, 9]. Pelnac consists of two layers: a porcine tendon-derived atelocollagen sponge layer and silicon film [10]; it serves as scaffolding for the ingrowth of fibroblasts and endothelial cells, which contribute to neodermis formation and thereby improve wound healing [11, 12]. However, at present, the application of artificial dermis requires a second skin graft operation, which increases patients’ discomfort. We speculated that since artificial dermis mimics the scaffold structure of allogenic skin, it can be used as an overlay of micrografts in the same manner as allogenic skin to repair the wound in a single operation, while also promoting dermal and epidermal regeneration and collagen organization.

To investigate the above possibility, in the present study, we used Pelnac overlaid on minced split-thickness skin grafts to repair full-thickness skin defects in a rat model. The effectiveness of this treatment method was assessed by measuring the rate of wound healing; examining the formation of dermis and basement membrane; and evaluating the synthesis of collagen I and III, cell proliferation, angiogenesis, expression of growth factors related to wound healing, and scarring.

## Methods

### Reagents and antibodies

Antibodies against collagen I (cat. no. ab34710; Abcam), III (cat. no. ab7778; Abcam), and IV (cat. no. ab6586; Abcam); Ki67 (cat. no. ab15580; Abcam); cluster of differentiation (CD) 31 (cat. no. ab182981; Abcam); transforming growth factor (TGF)-β1 (cat. no. ab92486; Abcam) and TGF-β3 (cat. no. ab15537; Abcam); α-smooth muscle actin (α-SMA) (cat. no. ab32575; Abcam); vascular endothelial growth factor (VEGF) (cat. no. ab46154; Abcam); glyceraldehyde-3-phosphate dehydrogenase (GAPDH) (cat. no. ab181602; Abcam); and secondary antibodies [goat anti-rabbit IgG H&L (horseradish peroxidase) antibody (cat. no. ab97051; Abcam) and goat anti-mouse IgG H&L (horseradish peroxidase) antibody (cat. no. ab6789; Abcam)] were obtained from Abcam (Cambridge, MA, USA). The Picro Sirius Red Stain Kit was from Solarbio (Beijing, China). All other reagents were from Sigma-Aldrich (St. Louis, MO, USA). GAPDH was used as a protein loading control.

### Preparation of minced split-thickness skin grafts

Figure 1

**Fig. 1:**
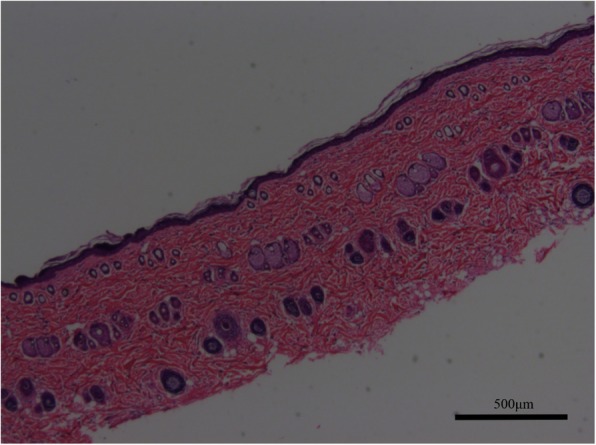
Biopsy of split-thickness skin graft of rats showing the thickness and structure (× 50). Scale bars, 500 μm

shows the histology of a biopsy of a split-thickness skin graft, including the thickness and structure. After propofol anesthesia, a Humby knife was used to harvest the grafts, which were then finely minced into micrografts using a standard scalpel blade. The specimens were placed in sterile saline to prevent their drying.

### Animal treatment

**Fig. 2:**
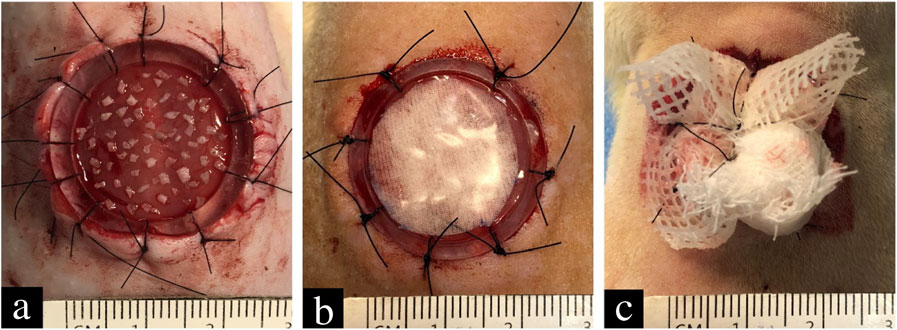
Micrografts transplanted into full-thickness skin defects of rats and covered with Pelnac or Vaseline gauze. **a** Prepared micrografts were transplanted into the full-thickness wound in the back of a rat at a 1:5 expansion ratio. **b** In the experimental group, the micrograft was covered with Pelnac. **c** Local packing and compression were applied to prevent graft displacement

This study was approved by the Ethics Committee of the Second Military Medical University Experimental Animal Center, and animals were handled according to international animal welfare standards. Male Sprague-Dawley rats of 10 weeks old were purchased from the Animal Center of the Second Military Medical University and individually housed under standard conditions in plastic cages. The animals were randomly divided into control (Vaseline gauze) and experimental (Pelnac) groups (*n* = 12 each). Hair removal and skin preparation were performed 1 day before the experiment. After anesthetization with propofol, a pen was used to draw a circle with a diameter of 20 mm in the middle of the back of each rat. Split-thickness skin grafts were obtained with a Humby knife from the circled area. Full-thickness skin was cut with eye scissors to create a circular full-thickness skin defect with a diameter of 20 mm; a sterile polymethyl methacrylate ring (outer and inner diameters of 20 and 18 mm, respectively) was then sutured to the inner edge of the wound to prevent its contraction. The prepared micrografts were transplanted into full-thickness wounds at a 1:5 expansion ratio, with the epidermis facing upward (Fig. 2a)

. The wound in the experimental group was covered with Pelnac (Fig. 2b) and that in the control group was covered with four layers of Vaseline gauze and one layer of silicon membrane for moisturizing. After local packing and compression to prevent displacement (Fig. 2c), the outer layer was wrapped with gauze.

### Measurement of healing rate

On postoperative days 7, 14, and 21, the wounds were photographed with a ruler alongside them at different time points, and the wound area was assessed using Image-Pro Plus software (Media Cybernetics, Rockville, MD, USA). The healing rate was calculated with the following equation: [(original area − non-healed area)/original area] × 100% [13].

### Measurement of shrinkage rate

The wounds were photographed alongside a ruler on day 35, and the wound area was assessed using Image-Pro Plus software (Media Cybernetics, Rockville, MD, USA). Wound shrinkage rate was calculated as [(original area – epithelized wound area on day 35)/original area] × 100%.

### Hematoxylin-eosin and Masson’s trichrome staining

Skin tissue was fixed overnight at 4 °C in 4% paraformaldehyde then embedded in paraffin and cut into 5-mm sections that were stained with hematoxylin-eosin (HE) or Masson’s trichrome. Images were acquired with a digital camera (Nikon, Tokyo, Japan) at × 50 magnification.

### Picrosirius red staining

Skin tissue was fixed overnight at 4 °C in 4% paraformaldehyde and then embedded in paraffin and cut into 5-mm sections that were stained with Picrosirius red. Images were acquired with a digital camera at × 200 magnification. Collagen area in different fields in each section was analyzed using Image-Pro Plus software.

### Immunohistochemistry

Sections on glass slides were deparaffinized and rehydrated, and endogenous peroxidase was quenched by treatment with 3% hydrogen peroxide for 10 min. Non-specific binding was blocked with 1% bovine serum albumin for 30 min. Antibodies against the following proteins were applied overnight at 4 °C: Ki-67 (1:200), CD31 (1:500), VEGF (1:200), collagen IV (1:100), TGF-β1 (1:50), TGF-β3 (1:100), and α-SMA (1:300). Biotinylated secondary antibodies were then applied at 1:200 for 30 min, followed by incubation with horseradish peroxidase–streptavidin at 1:400 for 30 min. The signal was detected by reacting with diaminobenzidine for 3–5 min, and the sections were counterstained with hematoxylin, dehydrated, and coverslipped. Images were acquired at × 200 or × 400 magnification using a digital camera.

### Western blot analysis

Skin tissue samples were lysed, and proteins were denatured and then separated on a 10% polyacrylamide gel, and transferred to a polyvinylidene difluoride membrane that was incubated in Tris-buffered saline with 0.05% Tween-20 (TBST) containing 5% non-fat milk for 1 h at room temperature. The gel was incubated overnight at 4 °C with antibodies against collagen I (1:5000), collagen III (1:7500), VEGF (1:4000), TGF-β1 (1:250), TGF-β3 (1:200), and α-SMA (1:2500). After washing with TBST for 15 min, the membrane was incubated with horseradish peroxidase-conjugated goat anti-rabbit or anti-mouse antibody (1:3000) for 1 h at room temperature followed by washes with TBST for 20 min. Protein bands were detected by enhanced chemiluminescence, and band intensity was analyzed using Image-Pro Plus software.

### Statistical analysis

Data are expressed as mean ± standard deviation (SD). Differences between two groups were evaluated with a Student’s *t* test, differences between three groups were evaluated with a one-way ANOVA, and differences between two groups at different time points were evaluated with a repeated-measures ANOVA. A *p* value of  < 0.05 was defined as statistically significant.

## Results

### Pelnac overlaid on minced split-thickness skin grafts accelerates wound healing rate and reduces wound shrinkage rate

**Fig. 3:**
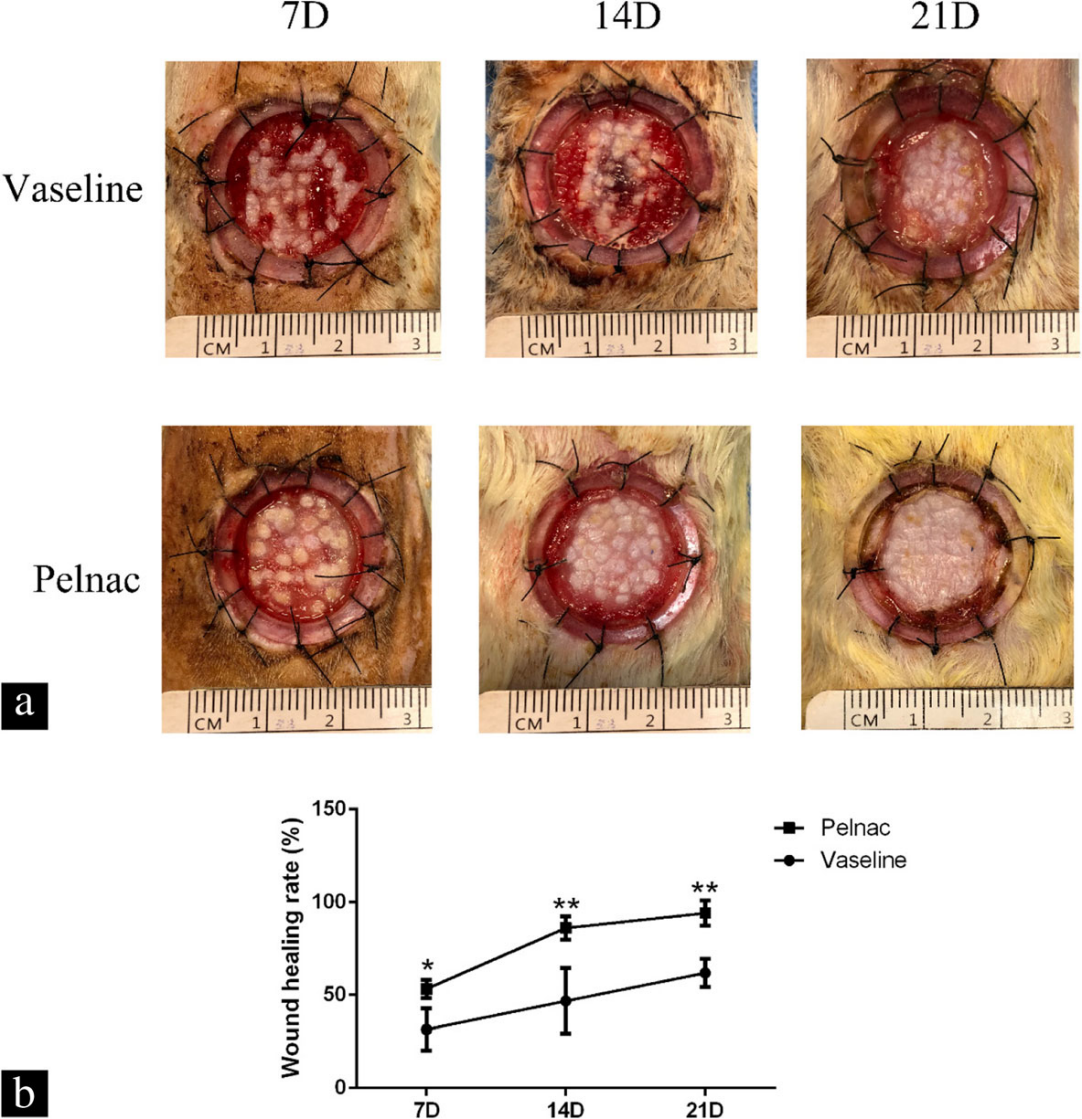
Wound size of rats at different time points post-operation. **a** Representative photographs of full-thickness skin wounds on days 7, 14, and 21 (7D, 14D, and 21D, respectively) after application of minced split-thickness skin grafts with Pelnac or Vaseline gauze as an overlay. **b** Statistical analysis of the wound healing rate in the two groups. 7D: Vaseline gauze group 31.51 ± 9.8%, Pelnac group 53.32 ± 4.3%; 14D: Vaseline gauze group 46.85 ± 15.3%, Pelnac group 86.19 ± 5.6%; 21D: Vaseline gauze group 62.00 ± 6.6%, Pelnac group 94.21 ± 5.9%. Data are presented as mean ± standard deviation. Error bars indicate standard deviation. Statistical analysis was performed by repeated-measures ANOVA. *p < 0.05, **p < 0.01

After 7 days of treatment, shedding of the Pelnac silica film was observed and the wound healing rate was higher in the Pelnac as compared to the Vaseline gauze group (*p* < 0.05). Similar trends were observed on days 14 and 21 (*p* < 0.01) (Fig. 3)

**Fig. 4:**
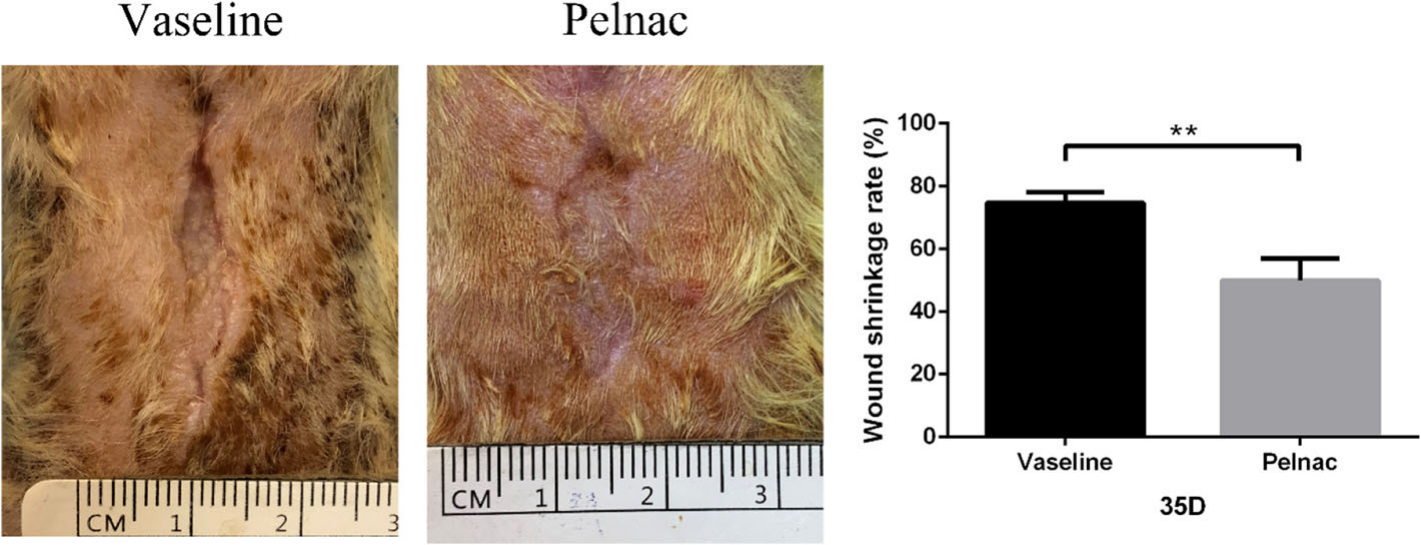
Wound shrinkage rate of rats on day 35 post-operation. Photographs of full-thickness skin wounds on day 35 after application of minced split-thickness skin grafts with Pelnac or Vaseline gauze as an overlay. Statistical analysis of the wound shrinkage rate in the two groups. Vaseline gauze group 74.78 ± 2.9%, Pelnac group 49.99 ± 6.1%. Data are presented as mean ± standard deviation. Error bars indicate standard deviation. Statistical analysis was performed by Student's *t*-test. **p < 0.01

. After 21 days of treatment, the polymethyl methacrylate ring (used for anti-contraction) loosened after contraction of the wound. After 35 days of treatment, the wounds epithelized completely in both groups but the shrinkage rate of the wounds in the Vaseline gauze group was higher than in the Pelnac group (*p* < 0.01) (Fig. 4).

### Pelnac stimulates dermis formation

**Fig. 5:**
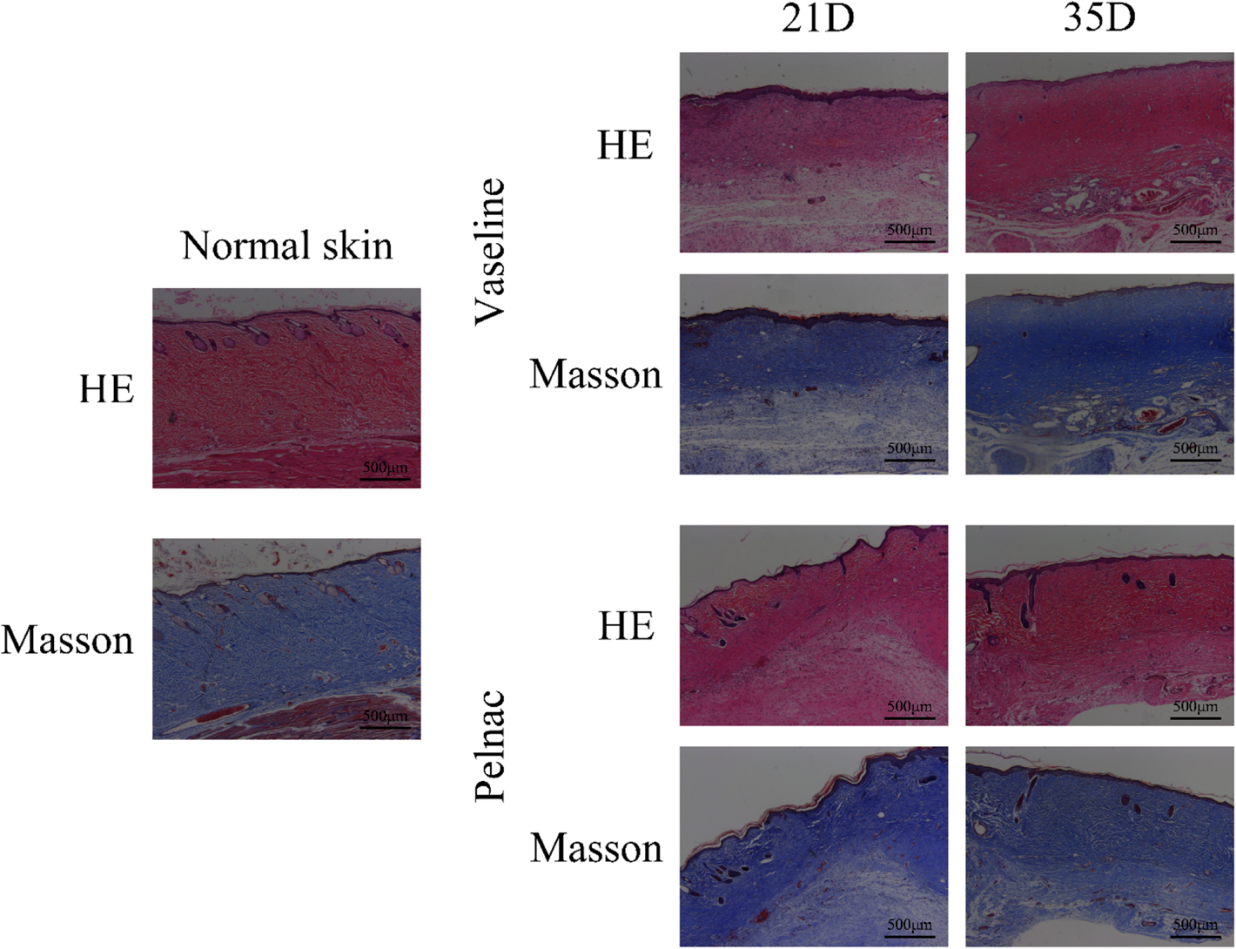
Formation of the dermis in skin wounds of rats at different time points post-operation. Staining performed by hematoxylin-eosin (HE) and Masson’s trichrome staining 21 days (21D) and 35 days (35D) after surgery in Vaseline gauze and Pelnac groups (× 50). Scale bars, 500 μm

A histological analysis by HE staining of wounds treated with Pelnac or Vaseline gauze overlaid on minced split-thickness skin grafts on postoperative days 21 and 35 revealed the complete formation of new epithelium on the wound surface (Fig. 5)

. Collagen is a major structural protein in the skin that is necessary for dermal tissue reconstruction at the wound site. We examined the wound tissue by Masson’s trichrome staining to detect collagen deposition on postoperative days 21 and 35. Compared to the Vaseline gauze group, an orderly arrangement of collagen fibers was observed in the Pelnac group, and the mature dermis had a thickness that was comparable to that of healthy skin.

### Pelnac increases basement membrane formation

**Fig. 6:**
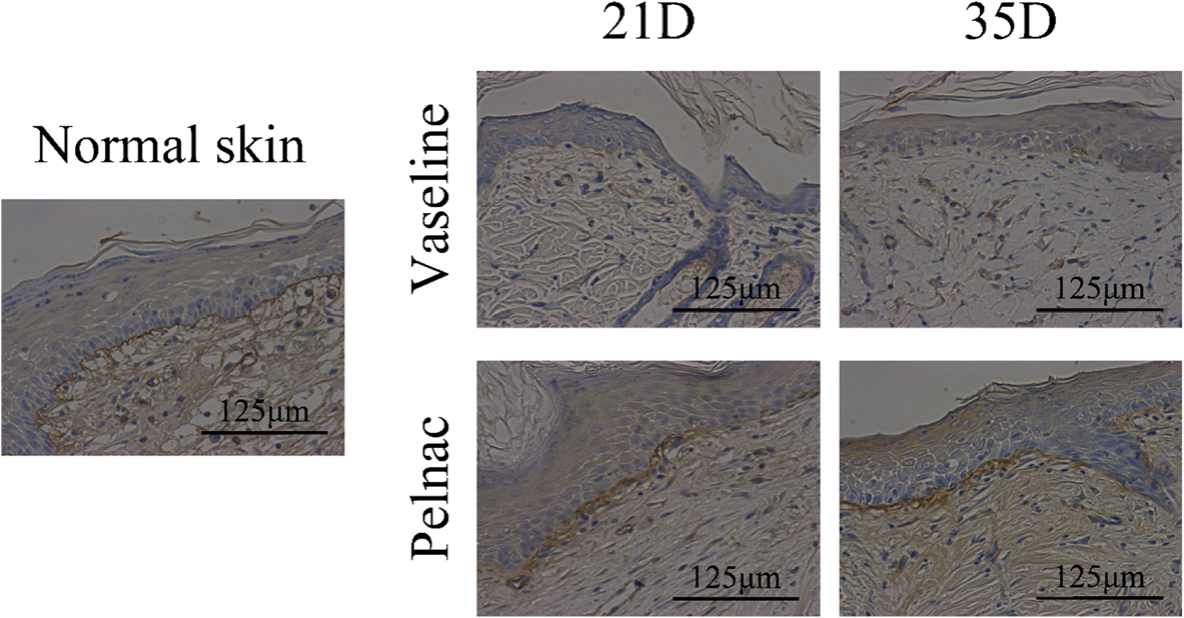
Type IV collagen deposition in the basement membrane of rats on days 21 and 35 post-operation. The deposition was monitored on day 21 (21D) and day 35 (35D), as determined by immunohistochemistry (× 400). Scale bars, 125 μm

The basement membrane is a connective tissue structure found in healthy skin tissue between the epidermis and dermis that mediates the interaction between these two layers and consists of a protein-rich matrix primarily composed of laminin and type IV collagen [14]. We examined the secretion and deposition of these proteins in the basement membrane of wound tissue by immunohistochemistry. Type IV collagen accumulation was observed in the basement membrane as a layer with thick, strong staining between the epidermis and the dermis (Fig. 6)

. The immunoreactivity was higher in the Pelnac group than in the Vaseline gauze group on postoperative days 21 and 35 and was comparable to that of normal skin at the later time point. The expression of these proteins in newly generated tissue reflects well-formed skin with a functional epidermis-dermis junction.

### Pelnac modulates collagen I and III synthesis

**Fig. 7:**
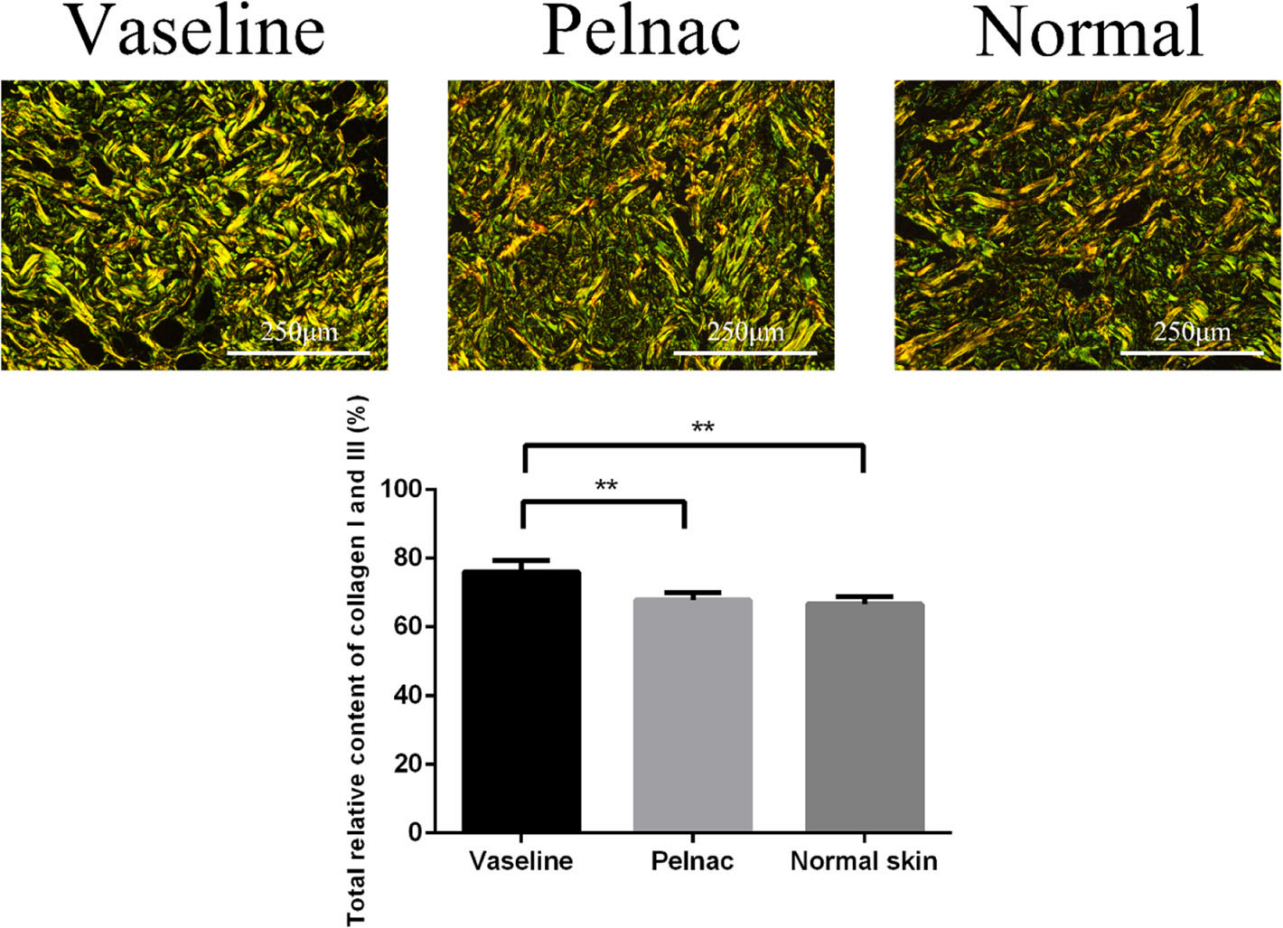
Total relative collagen Ι and III content in Vaseline gauze and Pelnac groups on day 35 post-operation. Data are representative of day 35 and are compared to normal skin. (Top) collagen I and III was detected by Picrosirius red staining (× 200). Scale bars, 250 μm. (Bottom) statistical analysis of total relative collagen Ι and III content. Vaseline gauze group 76.06 ± 3.0%, Pelnac group 67.78 ± 2.0%, normal skin 66.65 ± 2.1%. Data are presented as mean ± standard deviation. Error bars indicate standard deviation. Statistical analysis was performed by one-way ANOVA. *p < 0.05, **p < 0.01

Type I and III collagen are major components of the ECM and are required for tissue repair. However, excess collagen deposition can result in scarring. In order to evaluate the effects of Pelnac on ECM protein production, we assessed the expression of collagen Ι and III in tissue sections by Picrosirius red staining and the protein levels in tissue homogenates by western blotting on postoperative day 35. Total relative collagen Ι and III content was lower in the Pelnac as compared to the Vaseline gauze group (*p* < 0.01) and was comparable to that of normal skin (*p* > 0.05) (Fig. 7)

**Fig. 8:**
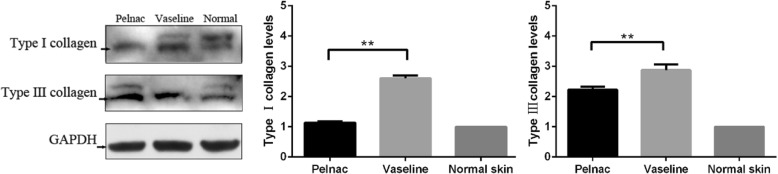
Pelnac as the overlay decreased collagen I and III expression in scars on day 35. (Left) collagen I and III levels in Pelnac or Vaseline gauze groups were detected by western blotting. (Middle and right) quantitative analysis of relative collagen I and III protein levels; the level in normal skin was set to 1. Data are presented as mean ± standard deviation. Error bars indicate standard deviation. Statistical analysis was performed by Student's *t*-test.*p < 0.05, **p < 0.01. *GAPDH* glyceraldehyde-3-phosphate dehydrogenase

. Similarly, the western blot analysis showed that collagen I (*p* < 0.01) and collagen III (*p* < 0.01) levels were lower in the Pelnac group than in the Vaseline gauze group on day 35 and collagen I was comparable to that in normal skin (*p* > 0.05) (Fig. 8)

. Thus, Pelnac prevents excessive collagen deposition and scar hyperplasia.

### Pelnac stimulates cell proliferation and angiogenesis in the skin

**Fig. 9:**
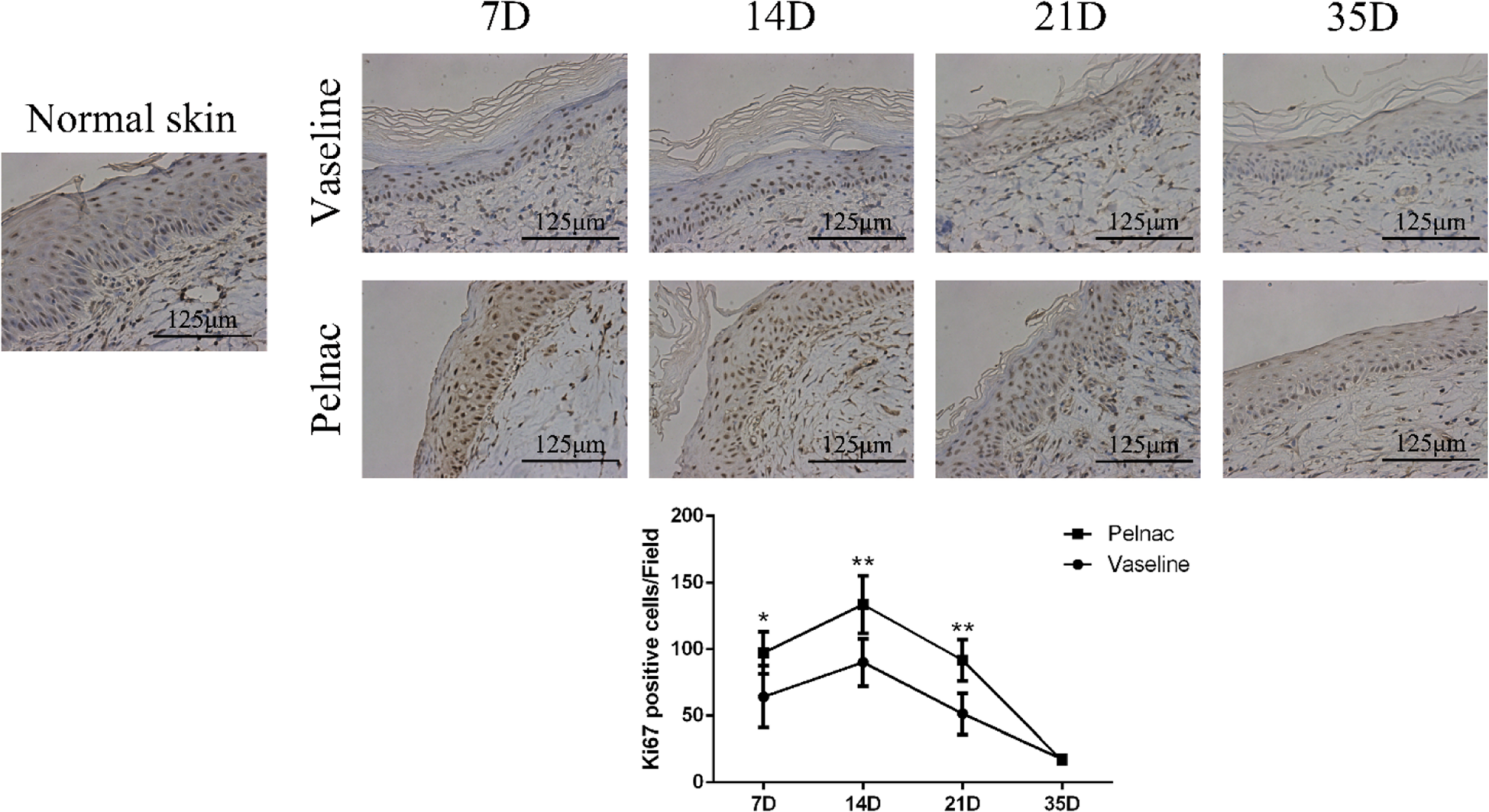
Ki-67 expression in wound tissue of rats at different time points post-operation after treatment with Vaseline gauze or Pelnac as overlay. (Top) Ki-67 immunohistochemistry was performed on days 7, 14, 21, and 35 (7D, 14D, 21D, and 35D, respectively) (× 400). Scale bars, 125 μm. (Bottom) quantitative analysis of Ki67-positive cells. Data are presented as mean ± standard devistion. Error bars indicate standard deviation. Statistical analysis was performed by repeated-measures ANOVA. *p < 0.05, **p < 0.01

The results of the immunohistochemical analysis revealed a higher number of Ki-67-positive cells in the Vaseline gauze and Pelnac groups than in healthy skin on days 7, 14, and 21, suggesting a compensatory increase in proliferation following injury. The number was significantly higher in the Pelnac as compared to the Vaseline gauze group on day 7 (*p* < 0.05), day 14 (*p* < 0.01), and day 21 (*p* < 0.01), suggesting that tissue repair was more active in the former group. Cell proliferate rate peaked on day 14 and was decreased on day 35 in both groups (Fig. 9)

**Fig. 10:**
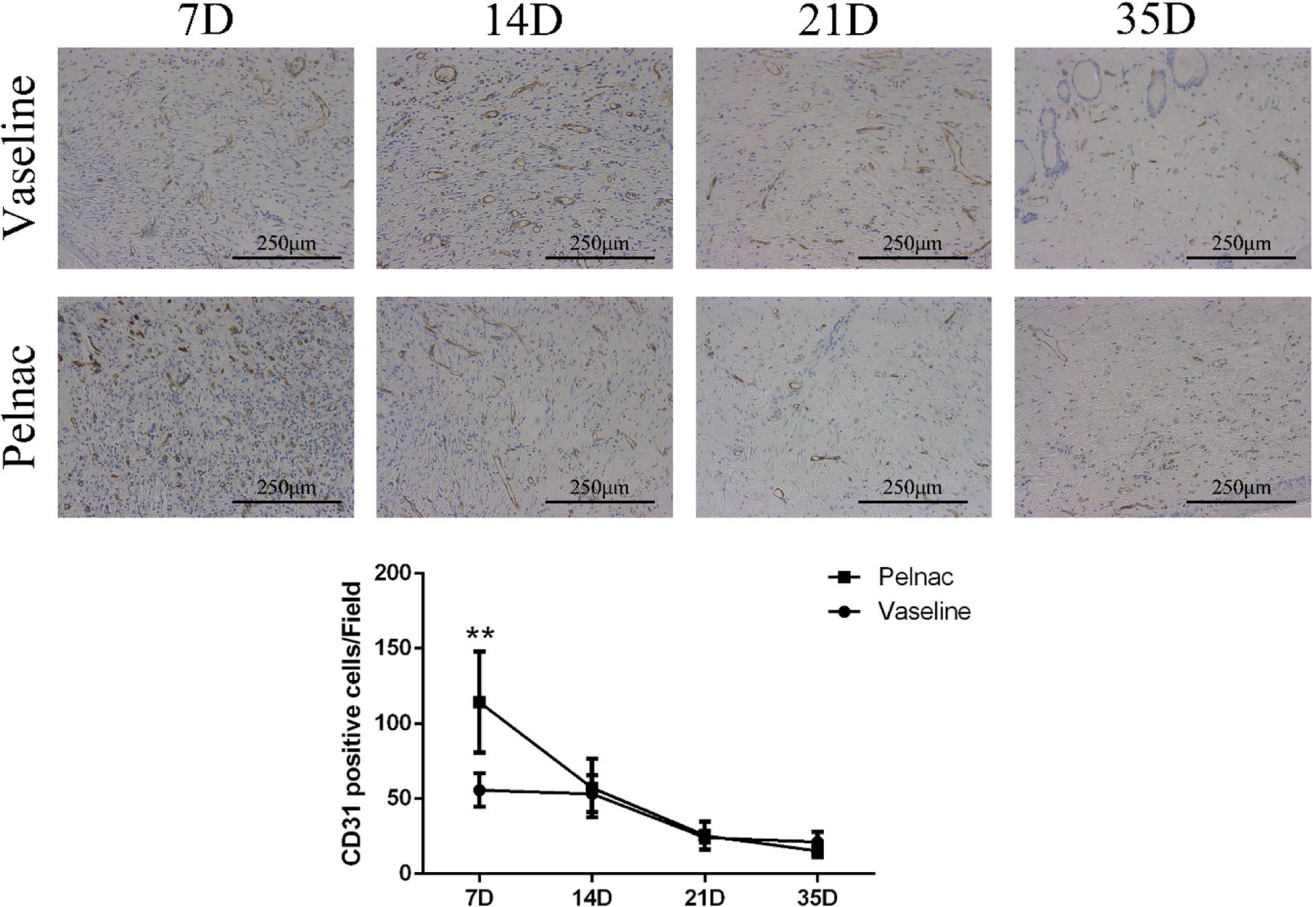
Cluster of differentiation 31 (CD31) expression in wound tissue of rats at different time points post-operation after treatment with Vaseline gauze or Pelnac as overlay. (Top) CD31 immunohistochemistry was performed on days 7, 14, 21, and 35 (7D, 14D, 21D, and 35D, respectively) (× 200). Scale bars, 250 μm. (Bottom) quantitative analysis of CD31-positive cells. Data are presented as mean ± standard devistion. Error bars indicate standard deviation. Statistical analysis was performed by repeated-measures ANOVA. *p < 0.05, **p < 0.01

. Similar dynamics were observed for the endothelial cell marker CD31: the number of CD31-positive cells was much higher in the Pelnac group than in the Vaseline gauze group on day 7 (*p* < 0.01), although there were no differences between the two groups on days 14, 21, and 35. The number of CD31-positive cells in both groups peaked on day 7 but was decreased on day 35 (Fig. 10)

. An analysis of capillary density [15] revealed comparable results. Thus, Pelnac promotes cell proliferation and neovascularization in skin tissue following injury.

### Pelnac decreases TGF-β1 and α-SMA expression and increases TGF-β3 expression in regenerated dermal tissue

**Fig. 11:**
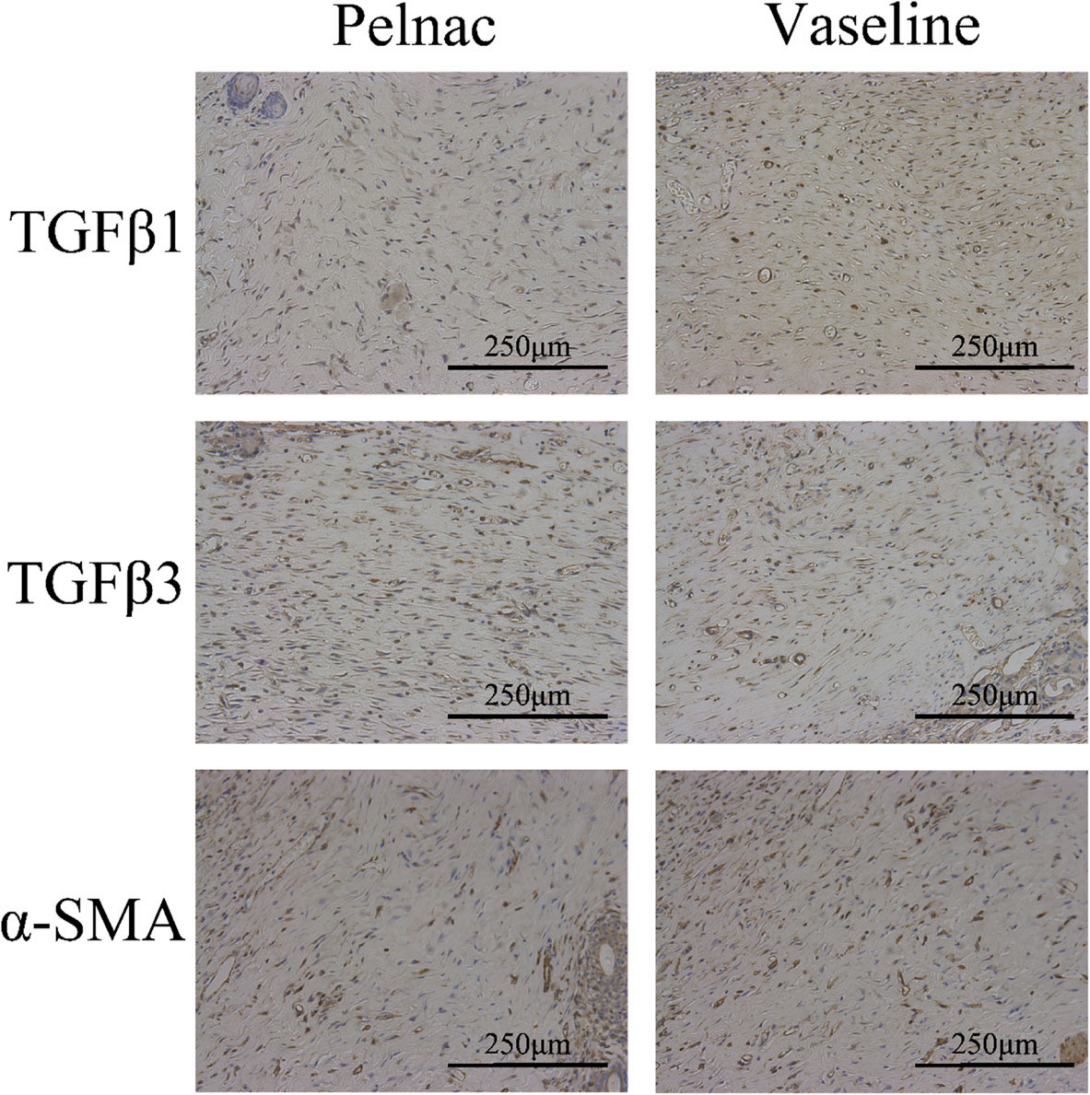
Transforming growth factor (TGF)-β1, TGF-β3, and α-smooth muscle actin (α-SMA) expression in wound tissue after treatment with Vaseline gauze or Pelnac as an overlay was detected by immunohistochemistry on day 35 post-operation (× 200). Scale bars, 250 μm

**Fig. 12:**
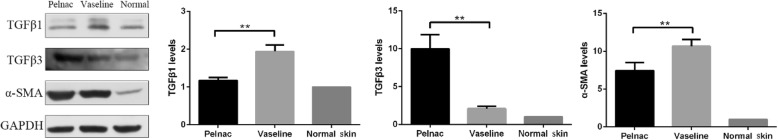
Pelnac decreases transforming growth factor (TGF)-β1 and α-smooth muscle actin (α-SMA) and increases TGF-β3 expression in regenerated dermal tissue on day 35 post-operation. (Left) TGF-β1, TGF-β3, and α-SMA levels following treatment with Pelnac or Vaseline gauze as overlay were detected by western blotting. (Middle and right) quantitative analysis of relative protein levels; the level in normal skin was set to 1. Data are presented as mean ± standard devistion. Error bars indicate standard deviation. Statistical analysis was performed by Student's *t*-test. *p < 0.05, **p < 0.01. *GAPDH* glyceraldehyde-3-phosphate dehydrogenase

TGF-β is known to be involved in wound healing; various TGF-β isoforms are implicated in different steps of the tissue repair process and scar formation. Hypertrophic scars in adults have elevated levels of TGF-β1, and suppressing TGF-β1 expression in wounds reduces scarring [16]. Other studies have suggested that elevated TGF-β3 levels and TGF-β3 to TGF-β1 ratio are critical for reducing scar formation [17]. TGF-β1 induces fibroblast differentiation into myofibroblasts, which express α-SMA [18], while α-SMA enhances wound contraction and scarring [19]. Based on these observations, we evaluated the effects of Pelnac on scar formation by immunohistochemistry and western blot analysis of TGF-β and α-SMA expression. Weak staining for TGF-β1 and α-SMA and strong staining for TGF-β3 were observed on day 35 in the Pelnac group (Fig. 11)

; western blotting indicated that TGF-β1 (*p* < 0.01) and α-SMA (*p* < 0.05) levels were lower whereas the TGF-β3 level was higher in the Pelnac as compared to the Vaseline gauze group on day 35 (*p* < 0.01) (Fig. 12).

### Pelnac enhances VEGF expression

**Fig. 13:**
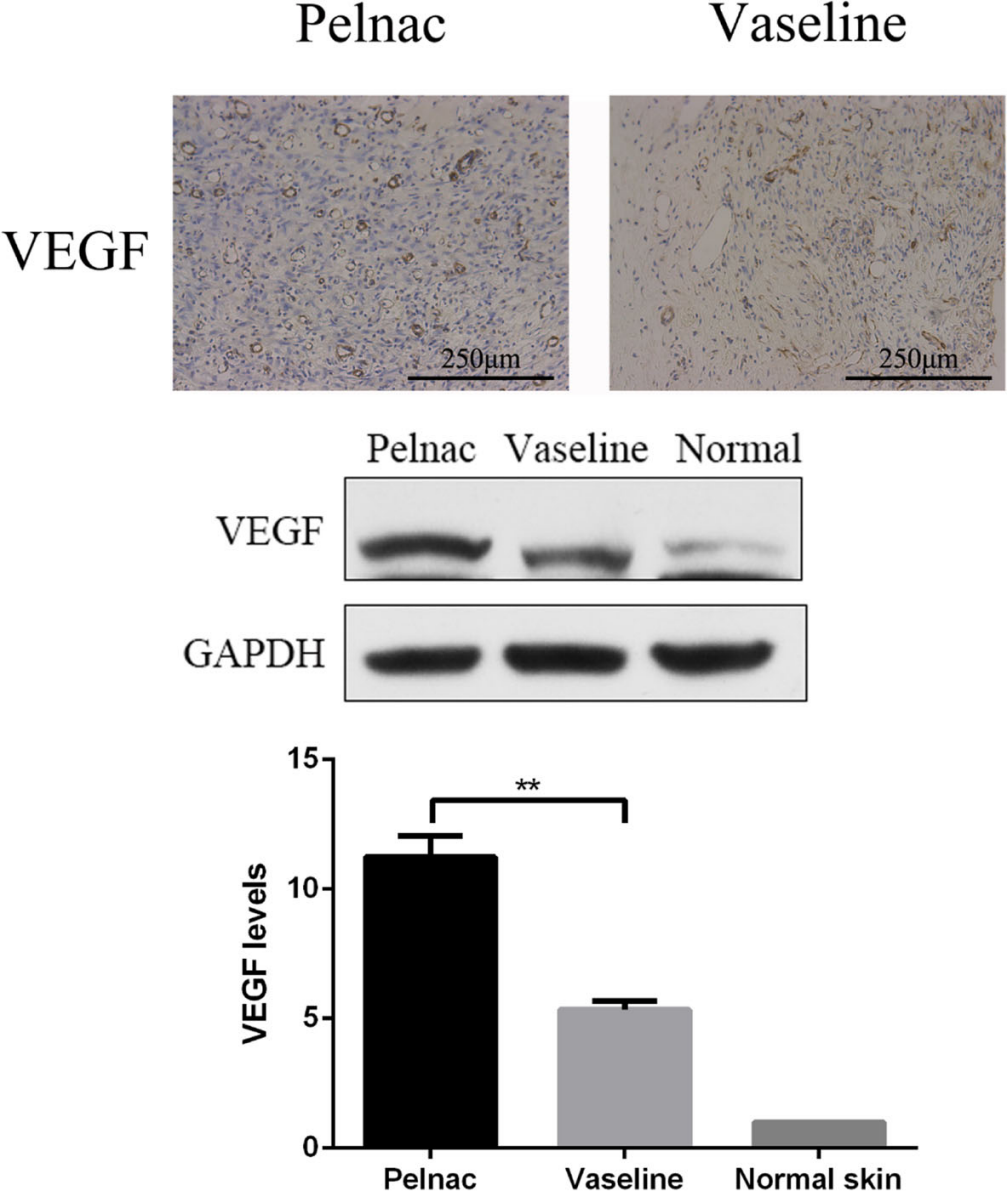
Pelnac increases vascular endothelial growth factor (VEGF) expression in rat wound tissue on day 7 post-operation. (Top) VEGF expression following treatment with Pelnac or Vaseline gauze as an overlay on day 7, as determined by immunohistochemistry (× 200). Scale bars, 250 μm. (Middle and bottom) VEGF expression following treatment with Pelnac or Vaseline gauze as determined by western blotting and quantitative analysis of expression levels. The level in normal skin was set to 1. Data are presented as mean ± standard devistion. Error bars indicate standard deviation. Statistical analysis was performed by Student's *t*-test.*p < 0.05, **p < 0.01

VEGF mediates angiogenesis, which plays an essential role in wound repair [20]. Based on our observation that vascularization was enhanced by Pelnac, we evaluated VEGF protein expression on day 7 by immunohistochemistry and western blotting and found that it was higher in the Pelnac group than in the Vaseline gauze group (*p* < 0.01) (Fig. 13).

### Re-epithelialization following minced split-thickness skin graft transplantation

**Fig. 14:**
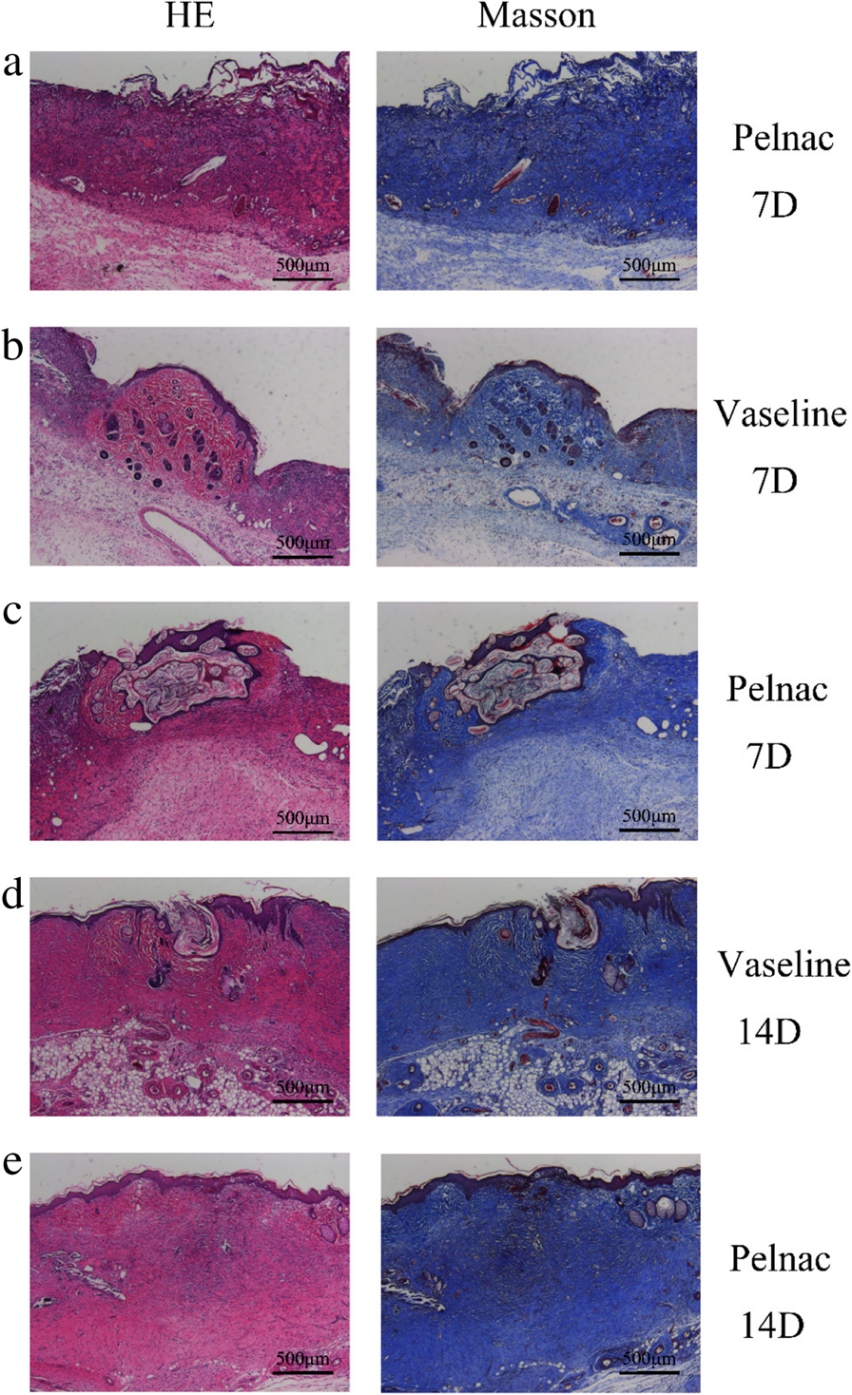
Effect of transplanted micrografts in full thickness skin defects with Pelnac or Vaseline gauze overlay on days 7 and 14. The figure shows Pelnac or Vaseline gauze overlay on collagen production in full-thickness skin defects on postoperative day 7 (7D) and day 14 (14D), as determined by hematoxylin-eosin (HE) and Masson’s trichrome staining (× 50). Scale bars, 500 μm. **a** Vascularization can be seen in the Pelnac group on day 7. **b** The minced split-thickness skin graft was attached to the wound base on day 7 in the Vaseline gauze group. **c** The minced split-thickness skin graft had reached the wound surface through Pelnac on day 7 in the Pelnac group. **d** The minced split-thickness skin graft had reached the wound surface on day 14 in the Vaseline gauze group. **e** The structure of the micrograft could not be seen in the Pelnac group on day 14

On postoperative day 7, Pelnac was undergoing the process of vascularization in the Pelnac group (Fig. 14a)

**Fig. 15:**
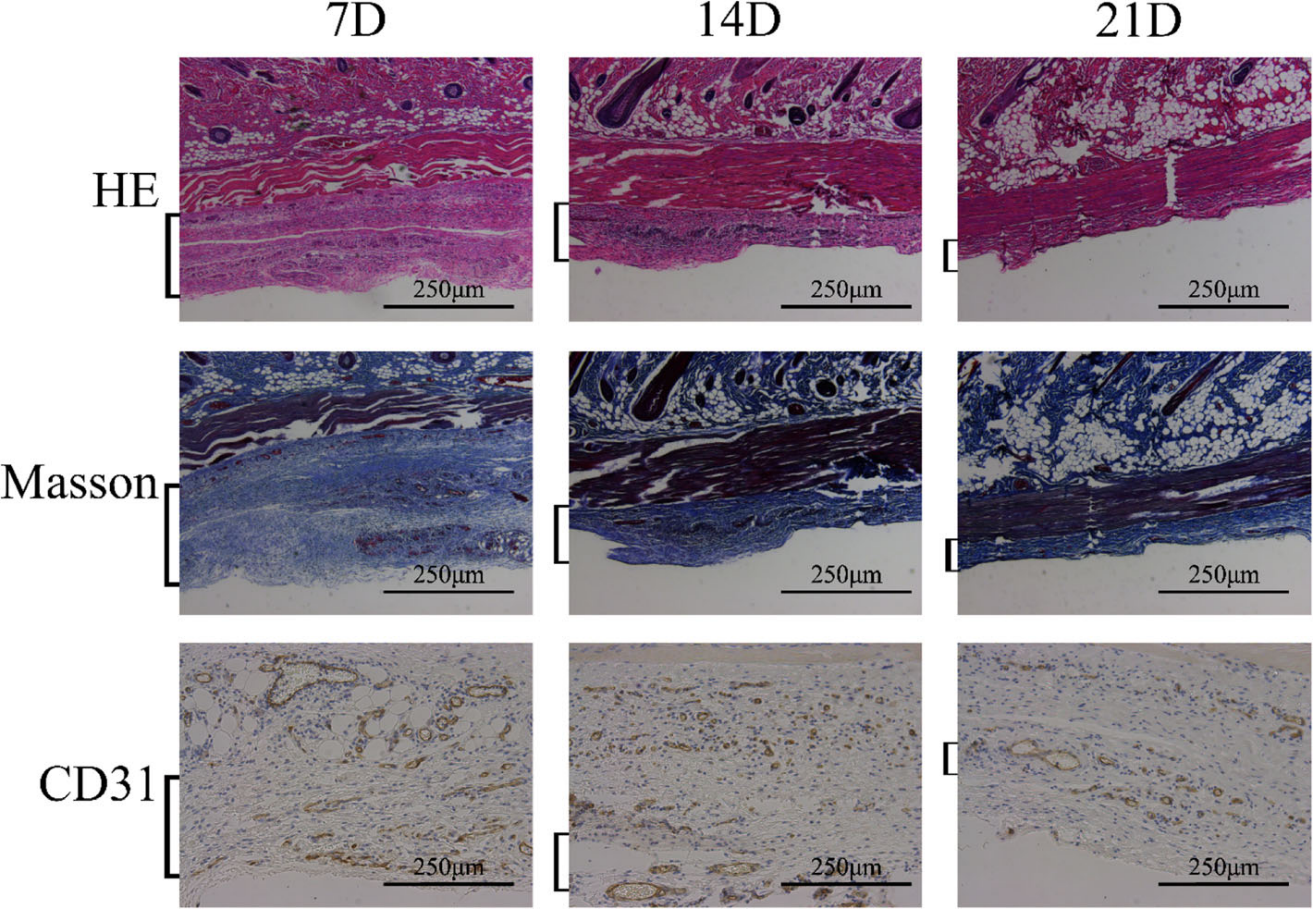
Vascularization of Pelnac after implantation under the dorsal skin of rats at different time points (square brackets). Vascularization after implantation under the dorsal skin of rats on postoperative days 7, 14, and 21 (7D, 14D, and 21D, respectively) visualized by hematoxylin-eosin (HE) and Masson’s trichrome staining and cluster of differentiation 31 (CD31) immunohistochemistry (× 200). Scale bars, 250 μm

. On postoperative day 7, thin granulation tissue had grown over the wound surface in the Vaseline gauze group, and the minced split-thickness skin graft was attached to the wound base (Fig. 14b) with no significant changes observed in the shape of the graft. In the Pelnac group, the cells from the graft had migrated to the wound surface through the Pelnac. During this process, the stratum corneum and transplanted dermis gradually separated from proliferating keratinocytes (Fig. 14c), but the same was not observed in the Vaseline gauze group until day 14 (Fig. 14d). After migrating from the base to the surface, keratinocytes appeared to expand radially in all directions and participate in re-epithelialization, as reported in previous studies [21]. On postoperative day 14, the micrograft structure was no longer visible in the Pelnac group; epithelialization was mostly completed, and the dermis had begun to form (Fig. 14e). In comparison, vascularization of Pelnac was observed on days 7, 14, and 21 after implantation under the dorsal skin of rats. Compared with the results shown in Fig. 10, we found that vascularization was more extensive when Pelnac was used as an overlay for minced split-thickness grafts than when it was implanted under the dorsal skin of rats (Fig. 15)

. At the same time, Pelnac was replaced by dermal tissue on day 21 after the operation. As the silicon film of Pelnac fell off naturally and there was almost no porcine tendon-derived atelocollagen sponge layer adhered to the silicon film when the wound was opened on day 7 after the operation, together with the fact that most of the Pelnac did not degrade in the rats for 7 days according to Fig. 15, we speculated that Pelnac was involved in dermal formation as a scaffold.

## Discussion

Promoting the rapid repair of full-thickness skin defects caused by severe burns and improving wound healing quality are significant challenges in burn treatment. The results of this study show that using minced split-thickness skin grafts with Pelnac as an overlay accelerates wound healing through enhanced cell proliferation and neovascularization. This treatment also stimulated the formation of a complete basement membrane connecting the epidermal and dermal layers of skin and alleviated scarring via modulation of collagen deposition and TGF-β expression.

Cell proliferation is essential for the repair and regeneration of damaged tissue. The expression of Ki-67, a marker of proliferation, was higher in the Pelnac group than in the Vaseline group, indicating that Pelnac may accelerate wound healing by providing a physical scaffold for proliferating cells. The local microenvironment of the wound also influences tissue repair. We found that the expression of VEGF—which regulates angiogenesis—was elevated in the Pelnac group, which could also promote wound healing. Pelnac also decreased and increased the expression of the scar-related growth factors TGF-β1 and TGF-β3, respectively, to minimize scar formation.

Disruption of the basement membrane leads to skin fragility and blistering [22]. The basement membrane and underlying dermis play critical roles in skin maturation and function by regulating keratinocyte growth and terminal differentiation [23, 24]. Deep burns can damage dermal tissue. Although transplantation of micrografts at a specific expansion ratio can enhance wound epithelialization, the basement membrane cannot be established. Without a connection between the new epidermal and dermal layers, the epidermis can readily blister and fall off, resulting in chronic ulcers. We found here that an intact basement membrane was present in the Pelnac group. Given that both keratinocytes and fibroblasts contribute to basement membrane formation [25–27], we speculate that Pelnac indirectly promotes basement membrane reconstruction by providing a scaffold for keratinocyte and fibroblast proliferation and migration. Indeed, dermal scaffolds can induce the formation of dermis-like granulation tissue, thereby improving the quality of wound healing [28]. HE and Masson’s trichrome staining of wound tissue on days 21 and 35 showed that the dermis was thicker and more ordered in the Pelnac as compared to the Vaseline gauze group. Pelnac also modulated the synthesis of collagen in the wound, thereby preventing excessive collagen deposition and hyperplasia. Full-thickness wounds close through re-epithelialization and contraction induced by α-SMA-positive myofibroblasts in granulation tissue; however, the persistence and activation of myofibroblasts during tissue regeneration lead to fibrotic scarring [29–35]. We observed that Pelnac suppressed α-SMA expression in the dermis; it may thus enhance ECM remodeling and accelerate wound closure independent of wound contraction by inducing fibroblast differentiation into myofibroblasts.

According to the healing process following minced split-thickness skin graft transplantation, we believe that Pelnac, as an overlay for minced split-thickness skin graft, still can complete the vascularization process and play its role as a dermal scaffold perfectly, and it does not affect the movement of minced split-thickness skin graft from the wound base to the wound surface; moreover, the minced split-thickness skin graft participates in the formation of the epidermis and dermis. However, as this was a rat model study for a duration of 35 days, there are some limitations. Further studies are needed to clarify how the interaction between minced split-thickness skin grafts and Pelnac promotes vasculogenesis and tissue regeneration.

## Conclusions

The results of this study demonstrate that using minced split-thickness skin grafts with Pelnac as an overlay is an effective one-step approach for the reconstruction of full-thickness skin defects in rats and can increase wound healing rate and quality. In addition to stimulating cell proliferation and angiogenesis, Pelnac modulated the synthesis and deposition of collagen and increased basement membrane and dermis formation along with the expression of growth factors related to wound healing while reducing scarring. This novel and promising treatment strategy can be applied to traumatic skin wounds caused by burns or injury.

## Abbreviations

CD: Cluster of differentiation
ECM: Extracellular matrix
HE: Hematoxylin-eosin
GAPDH: Glyceraldehyde-3-phosphate dehydrogenase
TGF: Transforming growth factor
VEGF: Vascular endothelial growth factor
α-SMA: α-Smooth muscle actin
